# Editorial: Caregiving for older adults within community settings

**DOI:** 10.3389/fpubh.2026.1804192

**Published:** 2026-03-03

**Authors:** Jodi L. Southerland, Steven A. Cohen, Roger O'Sullivan, Matthew Lee Smith

**Affiliations:** 1 Department of Community and Behavioral Health, East Tennessee State University, Johnson City, TN, United States; 2 Department of Public Health, University of Rhode Island, Kingston, RI, United States; 3 All Island Institute of Public Health (IPH), Dublin, Ireland; 4 The Bamford Centre for Mental Health and Wellbeing, Ulster University, Coleraine, United Kingdom; 5 School of Public Health, Texas A&M University, College Station, TX, United States; 6 School of Public Health, Indiana University, Bloomington, IN, United States

**Keywords:** caregiving, community, older adults, research, rural

## 1 Why this Research Topic matters

Globally, there is a growing demand for caregivers to support older adults within community settings, driven primarily by increased longevity. Global life expectancy is currently 73.5 years, with countries such as Japan, Switzerland, and the United Kingdom already surpassing 80 years. By 2050, life expectancy is projected to increase to 78.2 years (1, 2). At the same time, the number of individuals 65 years and older will more than double in size, increasing from 761 million in 2021 to 1.6 billion in 2050, with the fastest growth occurring among individuals aged 80 years and older (3). Disease patterns are also shifting from communicable to non-communicable diseases (NCDs) such as cardiovascular disease, cancer, diabetes, and dementia, which contribute to functional limitations, substantially increasing the need for caregiver support (1, 2, 4–6). In parallel with these changes, smaller family sizes, shifting migration patterns, and direct care workforce shortages are reshaping the caregiving landscape, further straining natural caregiving support. These factors are contributing to the unprecedented global demand for care, which outpaces the availability of caregivers (3). With as much as 80% of care being provided by unpaid caregivers and given the shortage of direct care workers (3), it is critical to understand the unique challenges faced by both to design tailored services, community resources, and policies that can enhance caregiver wellbeing, quality of care, and promote equity.

In this context, we initiated the Research Topic (RT) *Caregiving for Older Adults within Community Settings* to advance knowledge in this critical area of research.

## 2 Summary of the contributions

This Research Topic represents a collection of 28 papers authored by 180 contributors. Contributors are affiliated with 77 institutions and represent research conducted in eight geographic regions: China, Italy, Kazakhstan, Korea, Singapore, Spain, the Netherlands, and the United States. The Research Topic contains a variety of manuscript types, including 20 original research papers, three brief research reports, and one for each of the following submission types: clinical trial paper, community case study, systematic review, methods paper, and study protocol. Most of the original research papers (*n* = 18) used a cross-sectional study design. Of the 20, 16 analyzed quantitative data, including two that conducted secondary data analyses using large national datasets: the Behavioral Risk Factor Surveillance System (BRFSS) (Cohen et al.), and the Health Information National Trends Survey (HINTS) (Ahn et al.). Qualitative (*n* = 2) and mixed methods (*n* = 2) approaches round out the remaining original research papers. Across the entire Research Topic, 20 papers reported receiving funding support, and four of those were supported by the National Institutes of Health (NIH) in the United States.

The research in this Research Topic was conducted primarily with family or unpaid caregivers, although some papers were framed from the perspective of older adults or field experts. Three of the 28 papers involved research conducted among paid caregivers (Hwang et al.; Rong et al.; Smith et al.). This signifies a gap that must be addressed. Without increasing the evidence base and attention on the paid caregiving workforce, particularly the need for broad policy support and investments in training, equitable wages, career growth pathways, and support needs, the supply-demand gap and quality of care provided to older adults will worsen. Noteworthy is the number of appellations for caregivers in the Research Topic's titles. “Family caregiver” (*n* = 7) was the most widely used form, followed by “informal caregiver” (*n* = 5), “unpaid” (*n* = 3), and “dementia caregiver” (*n* = 3), and a few other variations. “Primary caregiver” was only referenced once (Hu et al.), which may indicate it is out of vogue. Standardizing terminology in caregiving research could reduce confusion and enhance the synthesis of findings.

Additionally, the Research Topic areas can be divided into five buckets: (1) paid and unpaid caregivers for people living with dementia (PLWD), (2) caregiver burden/strain/overload, (3) caregiver physical and emotional health, (4) service demand/use/capacity (e.g., community-based support; hospice care; long-term care; technology); and (5) training, educational, and support needs of caregivers. While 10 papers in the Research Topic addressed the rural context in varying degrees, from a brief mention in the results to a focused analysis, only three of those had a specific emphasis on rural populations as indicated in the paper's title (Betegón et al.; Santoyo-Olsson et al.; Savla et al.). We originally conceptualized this Research Topic to focus exclusively on low-resource and rural contexts. However, due to the limited number of submissions initially, we expanded the focus. While it is heartening that one-third of the papers included some focus on rural settings, future research is needed on the unique policy, systems, and environmental changes required to support paid and unpaid caregivers in these contexts.

## 3 Significance and impact

This Research Topic reinforces that caregiving of older adults within community settings is important for those individuals receiving care and those providing care formally and informally. Additionally, it highlights that caregiving is important to society because it helps support dignity, independence, social connection, health, and reflects and builds on the social contract that values older people as full citizens. The emphasis on rural-urban differences and global diversity across the studies further underscores the need to account for critical cultural and contextual factors that contribute to a wide array of caregiver and care receiver outcomes.

## 4 Future directions

While the articles within this Research Topic advance critical knowledge in the field of caregiving, several areas warrant further exploration to address gaps, challenges, and opportunities within this evolving landscape. Potential avenues of future research inspired by this Research Topic include the following, alliterated with three Ps:

- Populations of interest

Efforts are needed to examine caregiving from heterogenous perspectives, a suggestion previously echoed by others (7), and expanded upon here. Distinct groups of unpaid caregivers have been historically under-represented in research including racial/ethnic minorities, LGBTQIA+ caregivers, and caregivers in low-resource and rural settings. Additionally, given the shift in family dynamics and the growing prevalence of multi-generational households globally, greater emphasis should be placed on the experiences of youth caregivers, including grandchildren, as well as long-distance and non-relative caregivers. Research could also center on the lived experiences of solo agers, Super Agers (90+), and Centenarians (one of the fastest growing age segments globally) to seek to gain a better understanding about how these individuals navigate the complex caregiving landscape and from whom they receive care and/or provide care to, such as a child (downward caregiving), given the rise of chronic conditions and disability at younger ages than in previous decades (8). As mentioned earlier in this editorial, there is also an opportunity to examine caregiving through the lens of the paid caregiving workforce in clinical and home or community settings. Finally, little attention has been given to the “caring triad” (i.e., the unpaid caregiver, paid caregiver, and care receiver team), despite the growth of home and community-based services that enable this experience.

- Potential topic areas

Community and clinical settings are feeling the strain of inadequate preparation for supporting older adults and their families. The global age-friendly ecosystem and development of multisector plans for aging in the U.S. (9, 10) represent an exciting opportunity to center caregivers within these frameworks. Moreover, research that highlights the importance of age-friendly employers and universities in supporting unpaid caregivers would be beneficial (11). Comparison of national policy frameworks, such as the National Strategy to Support Family Caregivers (12), offers an opportunity to promote transnational learning. Other topic areas include caregiver navigation services, integration of family caregiving within clinical care models (13), caregivers' technology use [(7), Smith et al.] in clinical and community settings, AI and generative AI to support families and agencies in the aging network, and experiential learning opportunities for health profession-motivated high school students designed to enhance the paid caregiving workforce pipeline. Lastly, most studies in the Research Topic focused on systems- and individual-level challenges related to caregiving. More emphasis should be placed on describing successful approaches or frameworks for supporting caregivers, protective traits that drive caregiver wellbeing, and caregiver-friendly community success models, particularly in low-resource and rural contexts.

- Programmatic and methodological approaches

Co-design principles should be used to ensure that programs and policies are relevant, accessible, and aligned with caregivers' needs and preferences, thereby fostering more equitable outcomes (6). In these approaches, it is important to leverage implementation science to integrate existing caregiver evidence-based strategies into community settings (6, 7). Conducting longitudinal studies is essential to evaluating the impact of national frameworks, community-centered interventions (7), and training and support on caregiver-related outcomes. These strategies can uncover the effectiveness, economic returns on investment (ROI), and scalability of current approaches.

## 5 Conclusion

This Research Topic reflects the interdisciplinary nature and diversity of thought, topics, and methodological approaches used in caregiving research today. It offers a glimpse into the future by highlighting salient issues impacting caregivers and care receivers in different settings across the globe. With the growing emphasis on caregiving as a social determinant of health (14), this Research Topic can help fuel advancements in caregiving research and practice. Browning et al. (6) recently published an editorial entitled “Grand Challenges: Addressing the Global Challenge of Healthy Aging” in the Aging and Public Health Section of *Frontiers Public Health*. In addition to the points they raised, this editorial adds caregiving to the list of grand challenges. Rosalynn Carter reminds us that caregivers are the cornerstone of society. Therefore, we believe this is a timely and relevant compendium of papers that will support researchers, practitioners, and communities in creating caregiver-friendly environments. This Research Topic and editorial share our insights and suggestions about future directions that can advance scholarship in this important area of research.
